# Allometric scaling and social modulation of resting metabolic rate in a eusocial mammal (*Heterocephalus glaber*)

**DOI:** 10.1242/bio.062586

**Published:** 2026-06-24

**Authors:** Jack E. Thirkell, Chris G. Faulkes, Hana N. Merchant, James D. Gilbert, Nigel C. Bennett, Daniel W. Hart, Monica A. Daley, Steven J. Portugal

**Affiliations:** ^1^ Department of Biological Sciences, School of Life and Environmental Sciences, Royal Holloway University of London, Egham, Surrey, TW20 0EX, UK; ^2^ School of Biological and Behavioural Sciences, Queen Mary University of London, London, E1 4NS, UK; ^3^ Mammal Research Institute, Department of Zoology and Entomology, University of Pretoria, Pretoria 0083, Gauteng, South Africa; ^4^ Department of Zoology and Entomology, University of Pretoria, Pretoria 0083, Gauteng, South Africa; ^5^ Department of Ecology and Evolutionary Biology, University of California, Irvine, CA 9269, USA; ^6^ Department of Biomedical Engineering, Henry Samueli School of Engineering, University of California, Irvine, CA 92697, USA; ^7^ Department of Mechanical and Aerospace Engineering, Henry Samueli School of Engineering, University of California, Irvine, CA 92697, USA; ^8^ Department of Biology, University of Oxford, Oxford, United Kingdom, OX1 3SZ, UK

**Keywords:** Bathyergidae, Naked mole-rats, Respirometry, Subterranean

## Abstract

Resting metabolic rate (RMR) underpins energy allocation to survival, reproduction, and longevity, yet its drivers in highly structured social systems remain unclear. We measured RMR in 76 naked mole-rats (*Heterocephalus glaber*) from five colonies to test effects of body mass, sex, reproductive status, age, social rank, and colony stability in this eusocial mammal. Body mass was the strongest predictor of RMR. Nevertheless, absolute RMR values were far below those predicted for mammals of similar mass, confirming metabolic suppression in this strictly subterranean species. Social factors produced subtler patterns. Among non-breeders, males had slightly higher (but not significantly so) RMR than females despite similar body mass. Breeding females showed elevated RMR relative to non-breeding females, but not significantly so. Age and dominance rank did not influence RMR once body mass was controlled. Colony identity explained little variation overall, although one colony showed altered metabolic scaling after social conflict, suggesting colony-level metabolic plasticity associated with hierarchical instability.

## Teaser

**Summary:** Naked mole-rat resting metabolic rate is primarily determined by body mass, remains unusually low for mammals, and shows limited influence from sex, reproduction, or social rank, although colony instability may alter metabolic scaling.

## INTRODUCTION

Metabolism is often described as the currency of life (McNab, 2008). A widely used measure of metabolism is basal metabolic rate (BMR), which represents the minimum amount of energy an endothermic animal requires to sustain essential bodily functions while resting, fasting, non-reproductive, and within its thermal neutral zone (TNZ) (Brody, 1945; White and Seymour, 2003). When the entirety of these conditions for BMR cannot be met, resting metabolic rate (RMR) is typically used in its place; resting energy expenditure under more natural or flexible conditions (Portugal et al., 2016; Swanson et al., 2017). Differences in RMR among individuals can substantially influence key life-history outcomes, including survival, development, reproductive performance, and senescence (Burton et al., 2011; but see Arnold et al., 2021). Although body mass is the dominant predictor of metabolic rate in mammals, consistent with the well-established allometric relationship between energy expenditure and size (Speakman, 2005), a range of additional intrinsic and extrinsic factors also contribute to within-species variation. These include age (Fukagawa et al. 1990), sex (Buchholz et al., 2001), reproductive condition (Molnar and Schutz, 1997), social status (Røskaft et al., 1986), and group or population affiliation (Merchant et al., 2024a), all of which may interact with or mask the influence of body mass. Consequently, without careful consideration of these contributing factors, RMR – while widely used – can be an imperfect indicator of how energy is allocated, particularly in species with complex social systems and structured patterns of reproduction.

Subterranean mammals that exhibit cooperative breeding offer valuable systems for examining the relationships among social organisation, life-history traits, and physiological processes in shaping metabolic characteristics. The cooperatively breeding naked mole-rats (*Heterocephalus glaber*) are a particularly remarkable example: despite their small size and strictly underground lifestyle, they are exceptionally long-lived and displays a markedly lower metabolic rate than other mammals of similar body mass (Wilson, 1971; Bennett and Faulkes, 2000). The colonies of *H. glaber* are structured around a single reproductive female and a small number of breeding males, whereas most colony members function as non-reproductive workers of both sexes (Jarvis, 1981). These individuals forego reproduction and instead support the colony through activities such as burrow excavation, food collection, and colony defence (Gilbert et al., 2020; Toor et al., 2022). Such a social arrangement provides an uncommon framework for separating the effects of body mass from other influences on variation in RMR.

This study explores inter-colony variation in the RMR and the allometric scaling of RMR, across five entire colonies of *H. glaber*. On account of all of the animals being maintained under the same conditions, equally provisioned, and were assessed using the same methodological approaches, it was expected that, at the colony level, *H. glaber* would exhibit a comparable mean RMR. However, greater inter-colony variation in the allometric scaling of RMR was predicted, because *H. glaber* exhibits mass-dependent, dominance-based polyethism, and colonies differ in size and therefore in the proportional representation of behavioural roles. Colonies with a greater proportion of heavier defence-specialised individuals are expected to have relatively greater average musculature at a given body mass, which should elevate RMR and consequently produce a steeper scaling exponent.

## RESULTS

Colony-level body mass (g), RMR (ml O_2_ h^−1^) and respiratory quotient (RQ) data are presented in Table 1. Colony average RMR ranged from 37.9±9.8 ml O_2_ h^−1^ to 52.2±18.9 ml O_2_ h^−1^ (Table 1). Previous values of RMR for naked mole-rats ranged from 21.5 ml O_2_ h^−1^ to 92.4 ml O_2_ h^−1^ (Table 2). Variation in RQ was low within colonies (s.d. range from 0.06-0.1) and between colonies, with an average RQ for all individuals (*N*=76) of 0.7±0.1 (Table 1).

**Table 1.** Physiological measurements from five colonies of naked-mole rats

Colony	N	Body mass (g)	RMR (ml O_2_ h^−1^)	RQ
CF27	15	35.9±7.0	37.9±9.8	0.7±0.06
17A	19	33.6±15.0	37.9±15.5	0.7±0.06
G	9	45.8±7.6	52.2±18.9	0.8±0.07
B	25	49.2±9.4	50.0±21.4	0.7±0.1
11C	8	35.3±11.9	47.2±26.3	0.7±0.06
All	76	40.8±12.6	45.5±18.8	0.7±0.1

For measurement techniques, see Materials and Methods. RMR refers to resting metabolic rate (ml O_2_ h^−1^), and RQ to the respiratory quotient. Values presented are mean±s.d. RQ is presented with three decimal places due to the small nature of the s.d.

**Table 2.** Metabolic measurements from previous studies

Sample size	RMR (ml O_2_ h^−1^)	Study
14	21.45	McNab (1966)
19	42.80	Buffenstein and Yahav (1991)
20	36.1	Goldman et al. (1999)
4	48.4	Riccio and Goldman (2000)
24	44.2	Connor et al. (2002)
22	81.36	Kirby et al. (2018)
22	92.4	Pamenter et al. (2019)
76	45.5	This study

RMR and sample size from seven prior studies which reported the RMR of naked mole-rats.

### Body mass strongly predicts RMR across all individuals

**Fig. 1.**
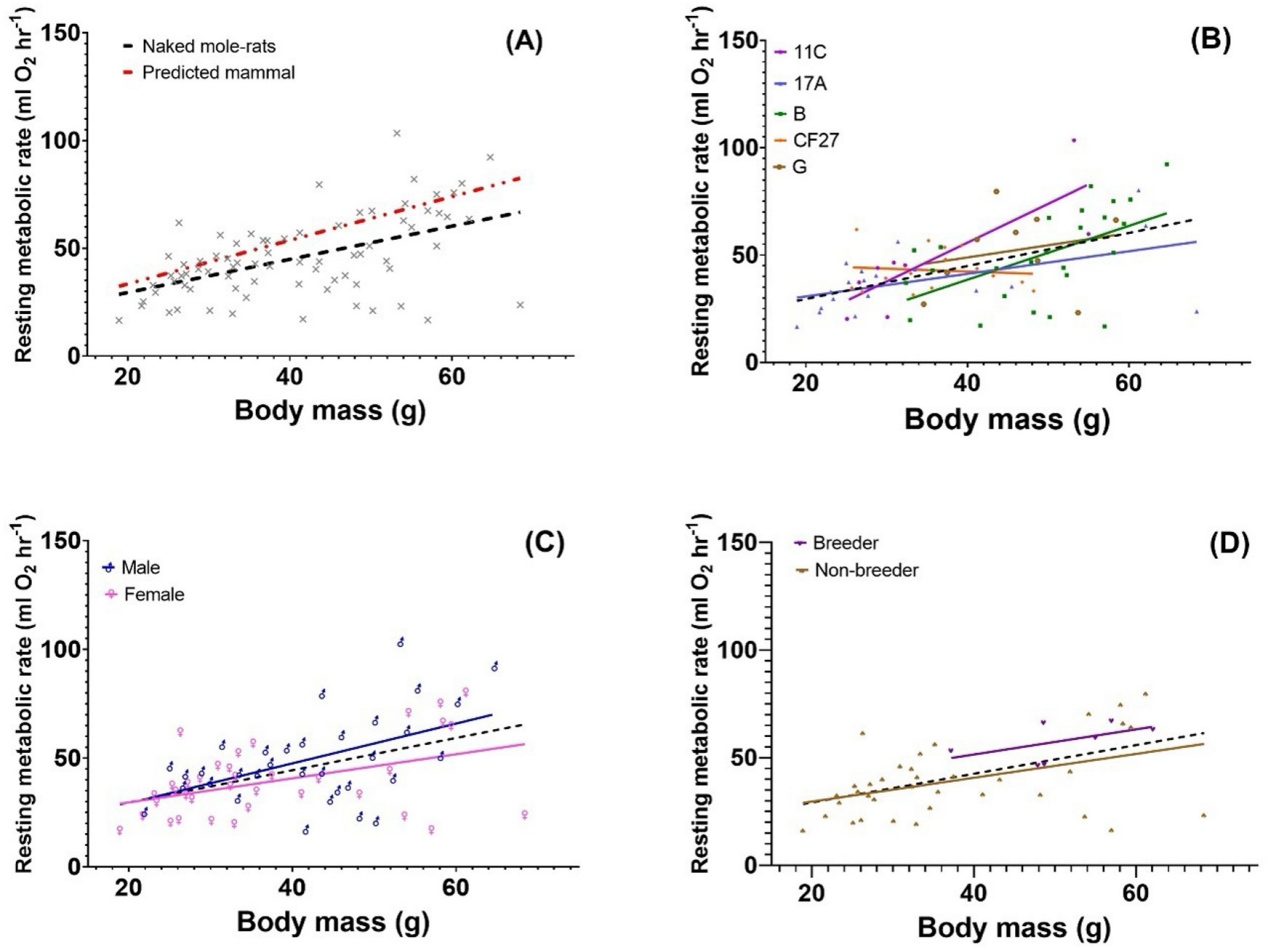
**Body mass and RMR (ml O_2_ h^−1^) in naked mole-rats (*H. glaber*).** (A) RMR–body mass relationship across 76 individuals. The black dashed line shows the fitted relationship for naked mole-rats, while the red dashed line represents the predicted allometric scaling for mammals (Kleiber's law: RMR=3.40×Body mass^0.75^). (B) RMR–body mass relationships for five colonies. Lines indicate colony-specific model fits. (C) RMR–body mass relationship for non-breeding individuals across sex (♀: Female; ♂: Male). (D) Female RMR–body mass relationship separated by breeding status (breeders versus non-breeders).

Resting metabolic rate increased significantly with body mass across the 76 naked mole-rats analysed (GLM: χ^2^_1_=26.56, *P*<0.001; Fig. 1A). Heavier individuals exhibited higher RMR. Model coefficients and additional parameters are reported in Table S1.

### Colony identity and interactions with body mass

**Fig. 2.**
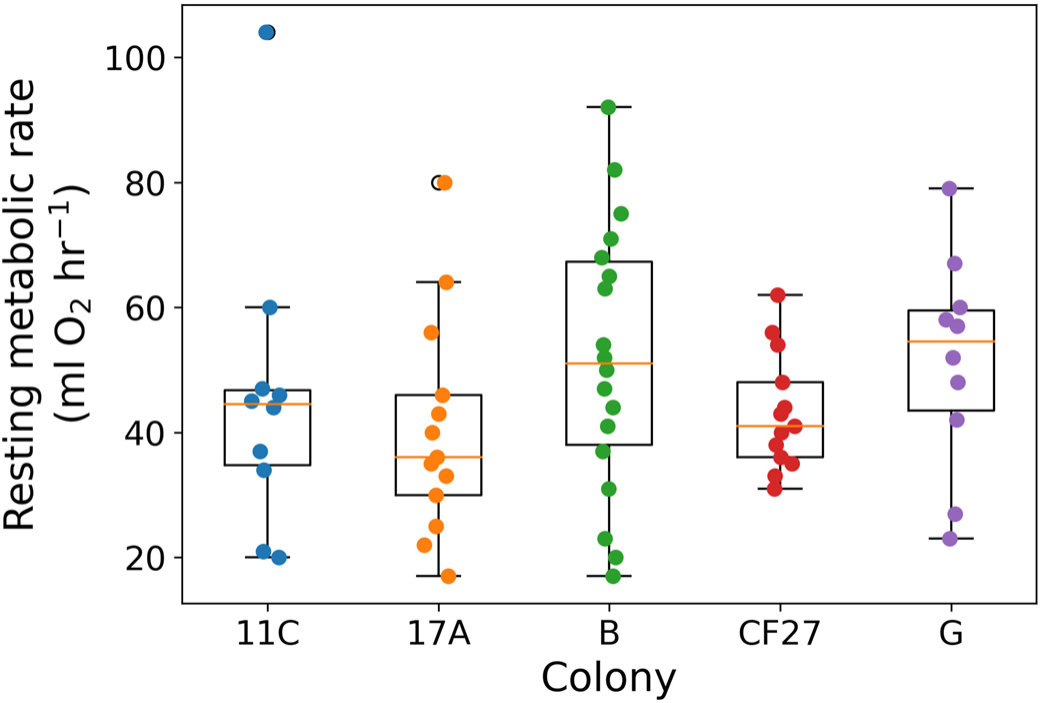
**The distribution of RMR (ml O_2_ h^−1^) in naked mole rats (*H. glaber*).** This includes the lower quartile (Q1), upper quartile (Q3), mean (open circle) and median (bold horizontal black line) values, for five entire colonies of naked mole-rats. No significant inter-colony differences in the mean RMR were determined (*P*>0.05).

When accounting for colony identity, body mass remained a significant predictor of RMR (χ^2^_1_=21.62, *P*<0.001), whereas colony alone did not explain significant variation (χ^2^_4_=2.24, *P*=0.69; Fig. 2). The interaction between body mass and colony approached significance (χ^2^_4_=9.42, *P*=0.05), suggesting slight variation in the body mass–RMR relationship among colonies (Fig. 1B). Full model coefficients are provided in Table S2.

### Sex differences among non-breeding individuals

In non-breeding mole-rats, RMR increased with body mass (χ^2^_1_=17.62, *P*<0.001), and there was a non-significant trend for sex differences, with males tending to have slightly higher RMR than females (χ^2^_1_=3.02, *P*=0.08). No significant interaction between body mass and sex was observed (χ^2^_1_=1.14, *P*=0.29; Fig. 1C). Model outputs are summarised in Table S3.

### Breeding status effects in females

Among females, RMR was positively associated with body mass (F_1,39_=9.74, *P*=0.003). Breeding status showed a nonsignificant trend, with non-breeding females having slightly lower RMR (F_1,39_=2.89, *P*=0.09), and the interaction between body mass and breeding status was not significant (F_1,39_=0.002, *P*=0.97; Fig. 1D). Full coefficients are provided in Table S4.

### Age-related effects

When controlling for body mass, age did not significantly affect RMR (GLM: χ^2^_1_=0.50, *P*=0.48; Fig. S1A). Body mass remained a strong predictor of RMR (χ^2^_1_=13.90, *P*<0.001). These results indicate that RMR differences among individuals are primarily driven by body mass rather than age. Model outputs are reported in Table S5.

### Social rank effects

In colonies with valid rank data, social rank did not significantly influence RMR once body mass was included in the model (GLM: χ^2^=1.61, *P*=0.21; Fig. S1B). Body mass remained the dominant predictor of RMR (χ^2^_1_=28.29, *P*<0.001). Model coefficients and dispersion parameters are provided in Table S6, confirming that variation in rank does not contribute to RMR differences after accounting for body mass.

## DISCUSSION

This study offers a detailed analysis of RMR in naked mole-rats, incorporating individual, social, and colony-level factors to clarify how energy expenditure is organised within a eusocial mammal. Body mass consistently emerged as the primary predictor of RMR, aligning with the well-established allometric scaling of metabolic rate across mammals. This follows widely accepted convention that body mass explains the majority of variation in mammalian metabolic rates (White and Seymour, 2003). In addition to the strong effect of body mass, we observed modest, yet biologically relevant differences linked to social and reproductive roles. Within the non-breeding group, males showed slightly elevated (but not significantly so) RMR compared with females. Crucially, this disparity is unlikely to result from sexual size dimorphism, as body mass did not differ significantly between non-breeding males (42.3±1.89 g) and females (37.5±2.27 g; *t*=1.63, *P*=0.11). There is also no consistent evidence for sex-based differences in activity levels that would account for variation in baseline energetic demands (Siegmann et al., 2021). Rather, the observed pattern may arise from subtle physiological or hormonal distinctions between the sexes, even under reproductive suppression. Overall, these findings indicate that pronounced reproductive skew does not entirely eliminate sex-related metabolic differences among subordinate individuals.

Reproductive role had an influence on metabolic rate. Breeding females showed higher RMR than non-breeding individuals, even when differences in body mass were accounted for (but not significantly so). This elevation likely reflects the considerable energetic requirements associated with ovulation, pregnancy, and subsequent lactation, and is consistent with findings in other mammalian species, where reproductive individuals maintain increased metabolic output to support these physiological demands (Schielke et al., 2017). That this difference remains evident under standard conditions suggests that reproductive effort produces a distinct metabolic effect that cannot be explained solely by overall body size or body mass. In a social system marked by extreme reproductive skew, such physiological specialisation may play a key role in sustaining caste-like functional distinctions. Queens are also much more active than non-breeders, with estimated double the activity and triple the distance travelled over continuous monitoring of a colony, compared with the next most active animal (C.G.F., personal observation).

Notably the absolute RMR values recorded here are substantially lower than those predicted for mammals of similar size (Table 3), further supporting the characterisation of naked mole-rats as possessing an unusually low metabolic profile. Predicted RMR values from 10 different studies and their associated approaches show a range of RMRs from 51.6 ml O_2_ h^−1^ to 71.1 ml O_2_ h^−1^, compared to an average RMR of 45.5 ml O_2_ h^−1^ in the present study. This metabolic depression is commonly viewed as an adaptation to their subterranean environment, where relatively stable burrow temperatures lessen thermoregulatory demands, and energetic efficiency is advantageous given the high energetic and water costs of excavation alongside constrained resource availability (Hart et al., 2022; but see also Merchant et al., 2024c). Within this ecological framework, reducing maintenance energy expenditure is likely to contribute both to colony stability and to the species' exceptional longevity.

**Table 3.** Predicted metabolic rate of naked mole-rats, assuming a body mass of 40 g

Reference	kcal/day	ml O_2_ h^−1^
Rubner (1883)	8.19	71.1
Kleiber (1932)	6.26	54.3
Brody (1945)	6.69	58.1
West et al. (1997)	6.26	54.3
Lovegrove (2000)	5.95	51.6
White and Seymour (2003)	7.87	68.3
Savage et al. (2004)	6.35	55.1
Glazier (2005)	7.12	61.8
McNab (2008)	6.59	57.2
Clarke and Rothery (2010)	6.80	59.0

Values converted to ml O_2_ per hour using the approximation 1 L O_2_ ≈4.8 kcal.

**Fig. 3.**
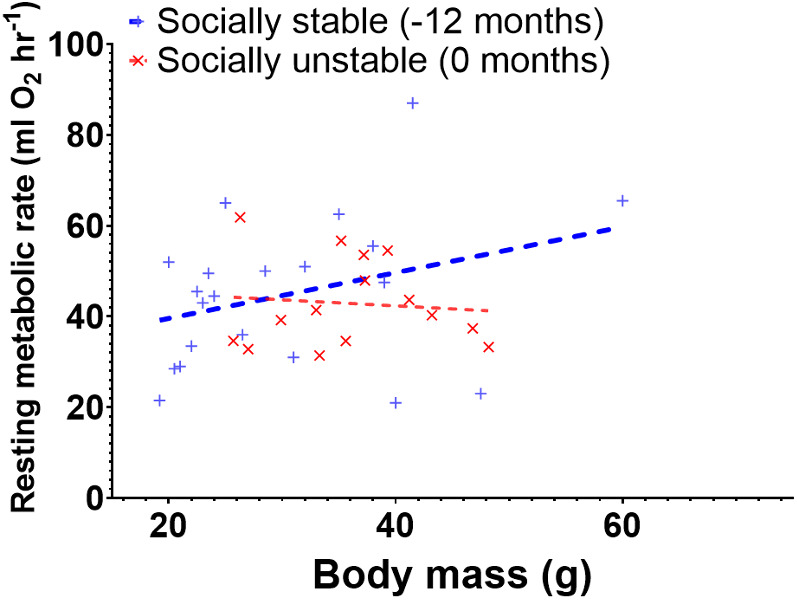
**Allometric scaling of RMR (ml O_2_ h^−1^) in colony CF27.** These measurements were taken during a period of colony instability (−12 months after first measurements; *N*=21) and following colony conflict and hierarchical instability (0 months; *N*=15) in naked mole-rats (*H. glaber*).

At the colony level, most groups conformed to expected allometric trends; however, notable deviations were observed. In particular, colony CF27 exhibited a shallower, and negative, body mass–RMR relationship, such that larger individuals expended less energy per gram than predicted. Strikingly, RMR measurements obtained from the same colony 12 months earlier yielded an allometric exponent comparable to other colonies in the present study (Fig. 3). Between sampling periods, CF27 experienced hierarchical instability, including aggressive interactions and the loss of individuals (larger animals likely involved in defence). We propose, therefore, that the altered scaling pattern reflects social disruption rather than measurement artifact. If confirmed, this would represent rare evidence of colony-level metabolic plasticity linked to social instability. Hierarchical disruption may alter reproductive function, stress physiology, task allocation, or dominance dynamics in ways that reshape energy allocation across body sizes. In obligately social species with strict dominance hierarchies, such plasticity could serve as an adaptive response to demographic perturbation, buffering colony-level energetic costs during periods of instability. More broadly, these findings raise the possibility that allometric scaling, often treated as a fixed biological rule, may exhibit socially mediated flexibility under certain ecological or demographic conditions.

In contrast, neither age nor dominance rank significantly predicted RMR once body mass was accounted for. The absence of an age effect is particularly notable given the exceptional lifespan of naked mole-rats. In many mammals, aging is accompanied by measurable shifts in metabolic maintenance; here, basal metabolism appears remarkably stable across age classes. This stability is consistent with the species' negligible senescence phenotype and suggests that aging does not impose detectable energetic costs at the level of resting metabolism. Similarly, the lack of a rank effect indicates that, although dominance determines reproductive access and social influence, it does not necessitate elevated resting energy expenditure. While it may be somewhat surprising that social rank did not influence RMR in this study, given that dominance-related differences in metabolic rate have been reported across a range of taxa (e.g. fishes, birds, and mammals) (Mathot et al., 2019), such effects are not universal. For instance, elevated metabolic rates in dominant individuals have been linked to increased activity, aggression, or the energetic costs of maintaining status in some systems, whereas other studies have found no clear association between dominance and baseline metabolic expenditure once activity and context are controlled (Mathot et al., 2019; Senar et al., 2000).

The absence of an effect in the present study may reflect several non-mutually exclusive factors. First, RMR was measured under standardised, resting conditions, which may minimise behavioural or physiological differences associated with social interactions that are more apparent in active metabolic rate. Second, dominance hierarchies in this species/context may be relatively stable or low-cost to maintain, reducing any sustained energetic differences between ranks. Third, environmental conditions (e.g. resource availability, group size, or housing conditions) can modulate the energetic consequences of dominance, potentially weakening rank–metabolism relationships.

Collectively, our results demonstrate that RMR in naked mole-rats is primarily governed by body mass, yet fine-scale variation emerges from sex, reproductive role, and colony-specific social dynamics. The species' markedly low metabolic baseline, combined with socially structured physiological modulation, highlights the tight integration of life history, ecology, and social organisation. Naked mole-rats, therefore, provide a powerful model for investigating how energy allocation strategies evolve under the dual pressures of cooperative living and extreme longevity. By maintaining exceptionally low maintenance costs while supporting complex social systems and extended lifespan, they illustrate how metabolic design can be optimised to meet the demands of eusociality. Understanding the mechanisms underlying this integration offers broader insight into metabolic evolution, the energetic costs of reproduction, and the physiological foundations of social complexity.

## MATERIALS AND METHODS

### Study animals

Captive populations (*N*=five colonies, *N*=76 individuals) of *H. glaber* (Rüppell 1842) were maintained in their respective colonies at the School of Biological and Behavioural Sciences, Queen Mary University of London. All mole-rats were captive born descendants of animals that were originally captured in Kenya during the 1980s and have a mixed genetic background because of originally crossing wild caught individuals from the north of Kenya with individuals from the south. Animals were identifiable, through subcutaneous radio-frequency identification (RFID) microchips and were marked with black marker pens to allow individuals to be distinguished during observations. Throughout this study, animals were provisioned with appropriate nesting material and were fed *ad libitum* on sweet potatoes, which were replaced daily. The animals were maintained in large polyurethane containers, housed in a climate-controlled laboratory that maintained an ambient temperature (T_a_) between 26-32°C, a relative humidity of 40-60%. Light changed unpredictably according (echoing occasional light exposure in the wild) to room use by research and facility staff during the day; lights were off for 12 h overnight. Animals were fasted for >4 h prior to assessments, to ensure a post-absorptive state and exclude the potential influence of digestion on metabolic activity (Šumbera, 2019). Despite an apparent absence of circadian rhythms among these species (Bennett and Faulkes, 2000), for continuity with other metabolic studies on African mole-rats and to follow established protocols, we conducted all assessments between 08:00-18:00 h, to mitigate against the potential effects of endogenous metabolic rhythms (Thirkell et al., 2025; Merchant et al., 2024b). Experimental procedures involving live animals and data collection described herein were approved by Royal Holloway University of London and Queen Mary University of London. The study was conducted in accordance with appropriate institutional and national guidelines.

### Social ranking

To test for an effect of dominance rank on working behaviour and aggression, we established the dominance hierarchy within each colony using passing behaviour, a reliable indicator of dominance relationships in naked mole-rats (Clarke and Faulkes, 1997). Specifically, we recorded instances in which one individual passed directly over another during face-to-face encounters within tunnels. Interactions not considered indicative of dominance included tail-to-face encounters, passing events occurring in chambers or tunnel corners, encounters in which one individual was digging throughout, and interactions where individuals did not pass directly over one another.

Individual dominance ranks were calculated using the Elo rating system implemented in the R package EloRating (Neumann and Kulik, 2020), as used previously for this study system (Gilbert et al., 2020). The Elo rating approach offers advantages over traditional matrix-based methods, including suitability for small group sizes and the ability to account for changes in group composition over time. To standardise ranks across colonies of varying sizes, individual Elo scores were scaled by dividing each rank by the total number of individuals observed in the respective colony. Lower scaled rank values (closer to zero) indicate higher dominance. We quantified the steepness of each colony's dominance hierarchy using the steepness function in the EloRating package, which derives values based on David's Scores (de Vries et al., 2006).

### Experimental procedure

Resting metabolic rate was determined through the measurement of the rate of oxygen consumption 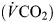 and carbon dioxide production 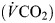, using an open-flow respirometer (Sable Systems International, Las Vegas, NV, USA) (see Thirkell et al., 2026 for further information on equipment). It has been suggested that for mole-rats, RMR may be a more appropriate measure of minimum energetic expenditure than BMR, on account of large interstudy disparities in the length of time animals have been fasted prior to metabolic assessment (Šumbera, 2019). Resting metabolic rate is considered to be between 10-25% greater than BMR, typically (Sherwood et al., 2005). Furthermore, for intrinsically social species, the stress associated with being separated for metabolic assessment has been noted to increase O_2_ consumption; *Fukomys damarensis* exhibited a 5% decrease, per individual, in O_2_ consumption when two individuals were assessed together (Wiedenová et al., 2018). It is for these reasons that RMR was considered an appropriate measure of energetic expenditure in this study.

Each respirometry assessment lasted a minimum of 65 min and consisted of a 10-min baseline to assess ambient O_2_ level, a 45-min metabolic assessment, followed by a further 10-min baseline to record ambient O_2_. Naked mole rats were kept and measured between 26-32°C, which is within the apparent thermoneutral zone of 27-34°C (Yahav and Buffenstein, 1991). The respirometer consisted of an airtight custom-made 2.5 l (19.2 cm long×15.2 cm wide×8.8 cm high) acrylic container, fitted with 4-mm inlet and outlet ports. Outside air (i.e. ambient air) was pulled through the respirometer at a flow rate of 300 ml min^−1^, resulting in a flush-out rate (63%) of approximately 8 min 20 secs, and 25 min (95%). The analogue outputs of O_2_ (%), CO_2_ (%), flow rate (ml min^−1^), relative humidity (%), barometric pressure (kPa) and temperature (°C) were recorded concurrently using a universal interface (UI2, Sable Systems International, Las Vegas, NV, USA). These measurements were sampled (1 Hz) and monitored in real-time using ExpeData software (Sable Systems International, Las Vegas, NV, USA), which enabled the progress and stability of each animal's respirometry trace to be visually assessed. Additionally, this enabled the manual addition of markers on the trace to note times of aberrant behavioural observations or external confounding factors. This real-time monitoring also safeguarded against potentially dangerous spikes in CO_2_ or drops in O_2_, at which point the assessment would have been terminated. Body mass (g) was measured immediately preceding each assessment using Oertling electronic weigh scales.

Incurrent airflow was controlled using a flow regulating pump (SS-4, Sable Systems International, Las Vegas, NV, USA), calibrated against a certified mass flow meter (FoxBox, Sable Systems International, Las Vegas, NV, USA), placed downstream of the respirometry chamber. Fractional concentration of O_2_ was measured using an oxygen analyser (FC-10a, Sable Systems International, Las Vegas, NV, USA), which was calibrated to ambient air O_2_ concentration (20.95%) before each trial. Fractional concentration of CO_2_ was measured using a carbon dioxide analyser (CA-10a, Sable Systems International, Las Vegas, NV, USA), and relative humidity measured using a water vapour analyser (RH-300, Sable Systems International, Las Vegas, NV, USA). Barometric pressure and temperature were measured from inbuilt sensors in the FC-10a oxygen analyser. Anhydrous Indicating Drierite™ was used to scrub atmospheric water from the excurrent air between the water vapour and CO_2_ analysers, and again between the CO_2_ scrubber and the oxygen analyser (W. A. Hammond Drierite Company LTD, USA) (see White et al., 2006). CO_2_ was scrubbed from the excurrent air between the CO_2_ and O_2_ analysers (Soda Lime, Sigma-Aldrich, Merck KGaA, Darmstadt, Germany).

Data, once exported from ExpeData, were processed in MATLAB (version 9.6. Natick, Massachusetts: The MathWorks Inc., 2019). O_2_ and CO_2_ were corrected for baseline drift and any time lag between these two variables (due to the delay in airflow between analysers) was corrected using cross-correlation. The fractional O_2_ signal was corrected for the removal of CO_2_ (O_2__corrected), the fractional CO_2_ signal was corrected for the removal of water vapour (CO_2__corrected), and the flow rate was corrected to Standard Temperature and Pressure (STP) conditions. A 5-min minimum analysis region was selected for RMR, corresponding to the lowest stable O_2_ consumption and CO_2_ production, during which the animal was considered to be most restful. The average over this period was used to obtain RMR estimates (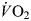 and 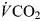), calculated using the formulae:

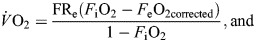


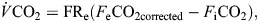
where *F*_I_ and *F*_e_ are incurrent and excurrent fractional concentrations (%) of O_2_ and CO_2_ (Lighton, 2008). The ratio of 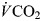 to 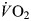 determined the respiratory quotient (RQ) (Lighton, 2008). For the purposes of data presentation mean RMR (ml O_2_ h^−1^) values were calculated from individual measurements of 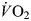 and are, unless otherwise stated, presented as the mean±s.d., corrected to STP conditions.

### Statistical analyses

All statistical analyses and figure generation were conducted in R (v4.5.2) and GraphPad Prism (v8.4). The dataset comprised 76 naked mole-rats from five colonies, with measurements including RMR, body mass (range: 18.9-68.4 g), age (range: 8-174 months), sex (male and female), breeding status (breeder and non-breeder), and social rank (0-1). Missing values in social rank were handled by: colonies with no rank data (*N*=2) were excluded from rank-related analyses, whereas individual missing ranks (*N*=1) were imputed using the midpoint between the minimum and maximum observed ranks within the respective colony. Animals with missing age data were excluded from analyses involving age (*N*=3 individuals).

Continuous variables (RMR, body mass, age, and rank) were assessed for normality using Shapiro-Wilk tests. Variables that met normality assumptions were analysed using linear models (LMs), while those violating normality assumptions were analysed using generalised linear models (GLMs) with Gaussian error distributions. Model outputs included regression coefficients, standard errors, *t-*values, and *P*-values (see Supplementary Material). The significance of main effects and interactions was evaluated using Type II ANOVA from the car package.

Separate models were fitted for each predictor to address specific biological questions. Body mass effects on RMR were first assessed across all individuals. Colony effects and interactions with body mass were then evaluated. Sex differences were examined among non-breeding individuals only, while breeding status effects were assessed in females, including interactions with body mass. Age-related effects were analysed using a model that included body mass as a covariate, ensuring age effects were independent of size. Similarly, social rank effects were assessed in colonies with valid rank data, with body mass included to control for size-dependent variation. Significance was interpreted using conventional thresholds (*P<*0.05), with trends reported for marginal *P*-values (0.05-0.10).

### Ethics

Experimental procedures involving live animals and data collection described herein were approved by Royal Holloway University of London and the Queen Mary University of London. The study was conducted in accordance with appropriate institutional and national guidelines.

## Supplementary Material



10.1242/biolopen.062586_sup1 Supplementary information

Table S1. Effect of body mass (g) on resting metabolic rate (RMR) across all individuals.
