# Supplementary material for: Allometric scaling and social modulation of resting metabolic rate in a eusocial mammal (Heterocephalus glaber)
Source: Biol Open. 2026 Jun 24;15(6):bio062586. doi: 10.1242/bio.062586 (PMC13382701; doi:10.1242/bio.062586)
Supplement: Supplementary information [file biolopen-15-062586-s1.pdf]

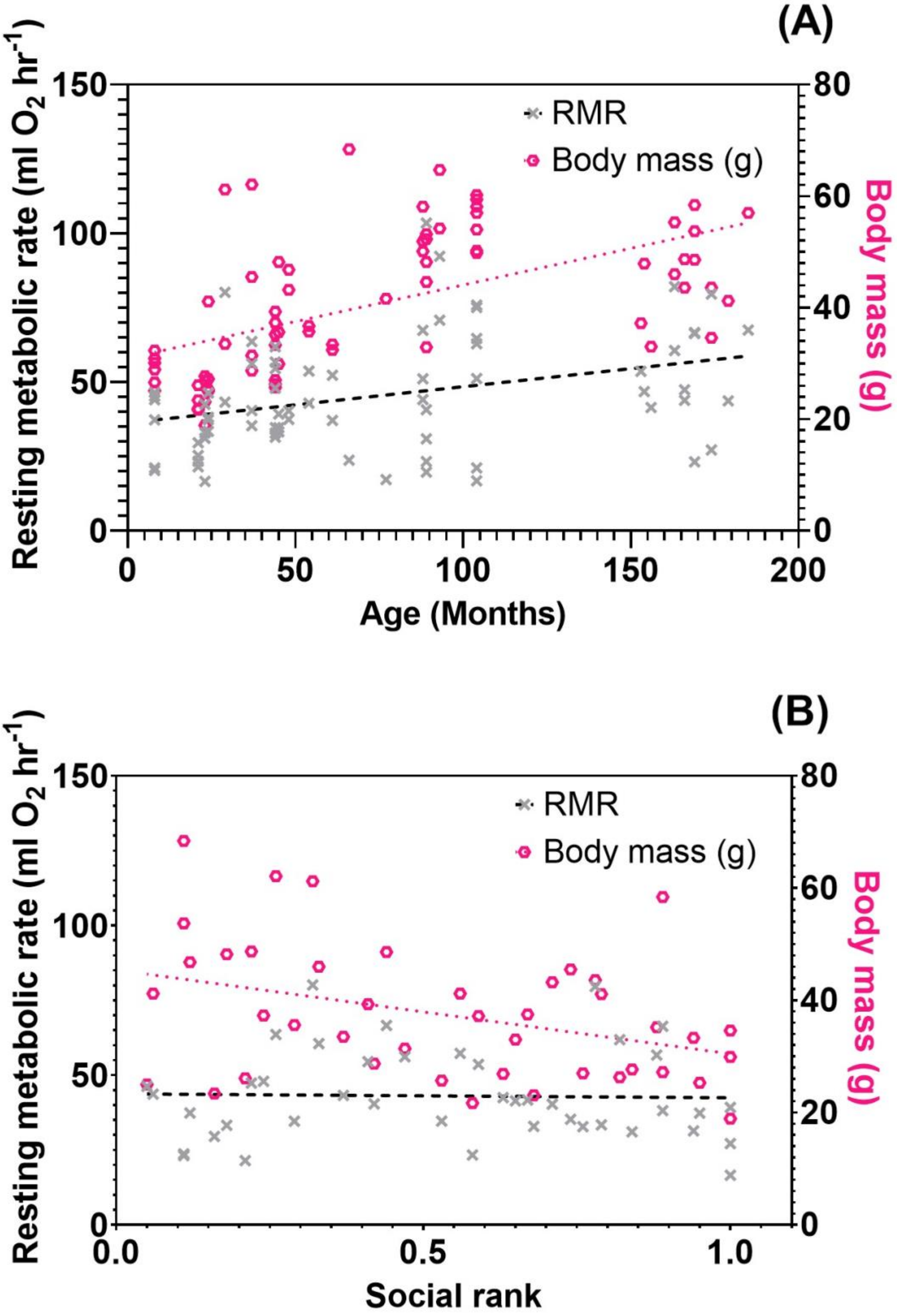

Fig. S1. Effects of age and social rank on resting metabolic rate (RMR, RMR, ml O_2 h^{-1}) in naked mole-rats (*Heterocephalus glaber*). (A) RMR in relation to age (black line) and body mass (pink line) across individuals with known age.(B) RMR in relation to social rank (pink line) and body mass (pink line) with valid rank data. Solid lines represent model predictions controlling for body mass.

Table S1. Effect of body mass (g) on resting metabolic rate (RMR) across all individuals.

Predictor	Estimate	Std. Error	*t* value	*P* value
(Intercept)	14.2	6.37	2.22	0.03
Body mass	0.77	0.15	5.15	<0.001*

Available for download at
https://journals.biologists.com/bio/article-lookup/doi/10.1242/bio.062586#supplementary-data

Table S2. Effect of colony (11C, CF27, 17A, G, B) and body mass (g) interaction on RMR.

Predictor	Estimate	Std. Error	*t* value	*P* value
(Intercept)	-16.6	18.7	-0.89	0.34
Body mass	1.81	0.51	3.58	0.001*
Colony17A	36.8	20.8	1.77	0.08
Colony B	4.97	25.5	0.20	0.85
Colony CF27	64.3	28.9	2.23	0.03*
Colony G	42.7	39.1	1.09	0.28
Body_mass:Colony17A	-1.28	0.56	-2.28	0.03*
Body_mass:ColonyB	-0.56	0.61	-0.91	0.37
Body_mass:ColonyCF27	-1.94	0.79	-2.47	0.02*
Body_mass:ColonyG	-1.24	0.90	-1.38	0.17

Table S3. Sex effects among non-breeding individuals and body mass (g) interaction on RMR.

Predictor	Estimate	Std. Error	*t* value	*P* value
(Intercept)	13.7	6.32	2.17	0.03
Body mass	0.68	0.16	4.19	<0.001*
Sex Male	7.20	4.14	1.74	0.08
Body mass:SexMale	0.25	0.23	1.14	0.29

Table S4. Breeding status (breeding and non-breeder) effects in females and body mass (g) interaction on RMR.

Predictor	Estimate	Std. Error	*t* value	*P* value
(Intercept)	28.2	38.6	0.73	0.47
Body mass	0.58	0.75	0.78	0.44
Breeding status Non-breeder	-9.59	39.2	-0.24	0.81
Body mass:Breeding statusNon-breeder	-0.03	0.77	-0.04	0.97

Biology Open • Supplementary information

Table S5. Age (months) effect on RMR, correcting for body mass (g).

Predictor	Estimate	Std. Error	*t* value	*P* value
(Intercept)	14.9	6.62	2.25	0.03
Age	0.03	0.04	0.71	0.480
Body mass	0.69	0.19	3.73	<0.001*

Table S6. Social rank effect on RMR, correcting for body mass (g).

Predictor	Estimate	Std. Error	*t* value	*P* value
(Intercept)	6.00	9.04	0.66	0.51
Rank	11.1	8.77	1.27	0.21
Body mass	0.83	0.16	5.32	<0.001*

Biology Open • Supplementary information
