# Supplementary material for: Allometric scaling and social modulation of resting metabolic rate in a eusocial mammal (Heterocephalus glaber)
Source: Biol Open. 2026 Jun 24;15(6):bio062586. doi: 10.1242/bio.062586 (PMC13382701; doi:10.1242/bio.062586)
Supplement: Supplementary file 1 [file biolopen-15-062586_review_history.pdf]

Allometric Scaling and Social Modulation of Resting Metabolic Rate in a Eusocial Mammal (Heterocephalus glaber)

Jack E. Thirkell, Chris G. Faulkes, Hana N. Merchant, James D. Gilbert, Nigel C. Bennett, Daniel W. Hart, Monica A. Daley and Steven J. Portugal
10.1242/bio.062586

Editor: Kendra J. Greenlee

Review timeline
Original submission: 24 March 2026
Editorial decision: 6 April 2026
First revision received: 26 May 2026
Accepted: 29 May 2026

Original submission

First decision letter

MS ID#: bio.062586

MS Title: Allometric Scaling and Social Modulation of Resting Metabolic Rate in a Eusocial Mammal (Heterocephalus glaber)

Authors: Jack E. Thirkell, Chris G. Faulkes, Hana N. Merchant, James D. Gilbert, Nigel C. Bennett, Daniel W. Hart, Monica A. Daley and Steven J. Portugal

I have now reached a decision on the above manuscript.

The reviewer reports are shown at the bottom of this email.

As you will see, the reviewers gave favourable reports, but raised some critical points that will require amendments to your manuscript. I hope that you will be able to carry these out, because we would like to be able to accept your paper. In particular, please ensure that statistically non-significant results are treated as such throughout the paper, including the abstract. In addition, it will be important to address the comments regarding definitions of BMR and SMR and how physical activity during measurements may have contributed to your findings.

At this stage, we also ask you to ensure your manuscript complies with our formatting guidelines – please see our manuscript preparation guidelines for details. Provided you are able to fully address the referees' comments, we are positive about publication of your paper (we accept over 95% of revision submissions) and therefore hope you won't mind any extra work involved in reformatting your manuscript at this point.

Please upload both a 'clean' version of your Word file, along with a highlighted version clearly showing where you have made changes in the revised manuscript. Please avoid using 'Track changes' in Word files as these are lost in PDF conversion.

I should be grateful if you would also provide a point-by-point response detailing how you have dealt with the points raised by the reviewers in the 'Response to Reviewers' box. Please attend to all of the reviewers' comments. If you do not agree with any of their criticisms or suggestions please explain clearly why this is so.

© 2026. Published by The Company of Biologists under the terms of the Creative Commons Attribution License (https://creativecommons.org/licenses/by/4.0/).

Reviewer 1

Comments for the author

This is a short and rather descriptive study reporting on careful measurements of metabolism in 76 individuals of naked mole rats belonging to five captive colonies. Metabolism was measured by closed respirometry. The authors suggest that their measurements reflect resting metabolic rather than basal metabolic rate. This is probably correct, but there is little attempt to account for the contribution of spontaneous physical activity during the measurements and it is also likely that any possible effects of sex, social status etc may be masked by the fact that resting metabolic rate is indeed higher than basal metabolic rate.

Specific comments
1. It may be worthwhile to introduce the definitions of BMR and SMR in the introduction, and to emphasise that reproducing females would violate the definition of BMR.
2. I am surprised that the idea that fossorial mammals are supposed to have lower metabolism (suggested by Brian McNab and others).
3. Metabolism has been measured numerous times in this species, and it would of interest to see a comparison to these earlier measurements. The impact of your paper could be strengthened by the inclusion of tabulated data and a brief discussion of differences/similarities.
4. The authors use intermittent closed respirometry. I think the duration of the flush period and the closed period could be described more firmly (lines 138-140) as 45 min plus 20 min (?).
5. The respirometry method must have result in 6-10 measurements per individual. Which was used to reflect SMR and how were these chosen. A typical approach is to choose the lowest measurements by some definition and the idea is to reduce the influence of spontaneous activity as much as possible. As mentioned above, I think the authors underestimate the contribution of spontaneous activity in these very active animals.
6. The description of the wash-out period is not correct (line 143); it takes much longer as the washout is a exponential and thus linger than volume of the divided relative flow. Normally that value results in 90% washout.
7. The authors describe measurements of CO_2 production but this data is not presented. It would be useful if RER was reported in more details
8. Lines 85- (and elsewhere). How different are the colonies in terms of the genetic background. In other words, are differences in metabolism to be expected and why?
9. Line 90. How does this temperature range compare to the thermoneutral zone of this species when kept under the conditions of the measurements (ie solitary with no possibility of huddling etc).
10. Lines 94-95. Are there any primary papers to be cited on the duration of the SDA response in mole rats?

Reviewer 2

Comments for the author

This study sets out to measure resting metabolic rate in individual naked mole-rats and correlate this to size, age, sex and social status. Five different colonies was studied and quite a lot of work must have gone into these experiments. As the authors point out, the social arrangement of naked mole-rats provides an uncommon framework for separating the effects of body mass from other influences on variation in RMR. The study appears to be professionally conducted and the manuscript is generally well written. However, I do have some comments.

The Introduction starts with the statement that "Resting metabolic rate (RMR) represents the baseline level of energy required to sustain essential physiological processes in endothermic animals and forms a central component of an individual's total energy expenditure". Does not that refer to basal metabolic rate (BMR) rather than RMR? For example, on page 5 it is stated that "Resting metabolic rate is considered to be between 10-25% greater than BMR, typically (Sherwood et al. 2005). Thus, the initial statement appears to be contradictory to what is later stated, studied and discussed.

© 2026. Published by The Company of Biologists under the terms of the Creative Commons Attribution License (https://creativecommons.org/licenses/by/4.0/).

Page 4. It is stated that "experimental procedures involving live animals and data collection described herein were approved by Royal Holloway University of London and Queen Mary University of London". Journals normally ask for an approval number so that the application can be traced.

Animals: I assume the animals must have been individually marked in some way to allow for the correlations to be made. How was this marking done?

On page 8 it is stated that "Breeding status showed a nonsignificant trend, with non-breeding females having slightly lower RMR ($F_{1,39}$ = 2.89, P = 0.097)". However, in the Abstract it is stated that "Breeding females showed elevated RMR relative to non-breeding females, consistent with reproductive costs.". Similarly, in the Discussion it is said that "Reproductive role had a stronger influence on metabolic rate. Breeding females showed higher RMR than non-breeding individuals". It seems misleading to talk about a "stronger influence" when it was not statistically significant. Please refer to this as a nonsignificant trend (like in the Results) also in the Abstract and Discussion.

Page 10. It is stated that "Notably the absolute RMR values recorded here are substantially lower than those predicted for mammals of similar size (Kleiber, 1932). Although Kleiber was a pioneer in this field, I suggest that the authors also cite some of the more recent and much more comprehensive papers on this topic.

Page 11. It is maybe surprising that social rank did not affect RMR. Such effects have been seen in other animals, from fish, to birds and mammals. This may be worth a bit of discussion and citations of studies finding/not finding dominance effects on RMR.

Fig. 2. The text and numbers on the axes are very small and hard to read. Please be consistent in the formatting of the figures.

Reviewer's Responses to Questions

Experimental quality
Does each figure have the proper controls?
If 'No', please indicate reasons in Comments for Author box below.
Reviewer #1:
- Yes

Reviewer #2:
- Yes

Were the data analyzed using appropriate statistical tests?
If 'No', please indicate reasons in Comments for Author box below.
Reviewer #1:
- Yes

Reviewer #2:
- Yes

Reproducibility
Were experiments performed using adequate number of biological replicates?
If 'No', please indicate reasons in Comments for Author box below.
Reviewer #1:
- Yes

Reviewer #2:
- Yes

© 2026. Published by The Company of Biologists under the terms of the Creative Commons Attribution License (https://creativecommons.org/licenses/by/4.0/).

Does the methods section provide sufficient detail to permit reproducibility?
If 'No', please indicate reasons in Comments for Author box below.
Reviewer #1:
- Yes

Reviewer #2:
- Yes

Completeness
Are the manuscript's conclusions supported by the data?
If 'No', please indicate reasons in Comments for Author box below.
Reviewer #1:
- Yes

Reviewer #2:
- Yes

Scholarship
Do the authors cite and discuss the merits of data that would argue for and against their conclusion?
If 'No', please indicate reasons in Comments for Author box below.
Reviewer #1:
- Yes

Reviewer #2:
- Yes

Does the manuscript title & abstract accurately reflect the contents of the manuscript, without hyperbole?
If 'No', please indicate reasons in Comments for Author box below.
Reviewer #1:
- Yes

Reviewer #2:
- Yes

First revision

<u>Author response to reviewers' comments</u>

<u>Reviewer 1:</u>

This is a short and rather descriptive study reporting on careful measurements of metabolism in 76 individuals of naked mole rats belonging to five captive colonies. Metabolism was measured by closed respirometry. The authors suggest that their measurements reflect resting metabolic rather than basal metabolic rate. This is probably correct, but there is little attempt to account for the contribution of spontaneous physical activity during the measurements and it is also likely that any possible effects of sex, social status etc may be masked by the fact that resting metabolic rate is indeed higher than basal metabolic rate.
Just to clarify that we used open open-flow respirometry, not closed, as stated in the Methods "*Resting metabolic rate was determined through the measurement of the rate of oxygen consumption ($\dot{V}O_2$) and carbon dioxide production ($\dot{V}CO_2$), using an open-flow respirometer (Sable Systems International, Las Vegas, NV)*".

© 2026. Published by The Company of Biologists under the terms of the Creative Commons Attribution License (https://creativecommons.org/licenses/by/4.0/).

"A 5-minute minimum analysis region was selected for RMR, corresponding to the lowest stable O_2 consumption and CO_2 production, during which the animal was considered to be most restful".
Please see below for responses to the other comments.

Specific comments:
1. It may be worthwhile to introduce the definitions of BMR and SMR in the introduction, and to emphasise that reproducing females would violate the definition of BMR.
SMR? Does the reviewer mean RMR (as we don't use term SMR)? We have now added the definitions of BMR and RMR into the Introduction, as suggested, and included the point that reproducing females (only the Queen, in this instance), would violate the definitions of BMR. Based on the comments from the other reviewer, we have re-written these sentences. This new text can be found below:

"Metabolism is often described as the currency of life (McNab 2008). A widely used measure of metabolism is basal metabolic rate (BMR), which represents the minimum amount of energy an endothermic animal requires to sustain essential bodily functions while resting, fasting, non-reproductive, and within its thermal neutral zone (TNZ) (Brody 1975; White and Seymour 2003). When the entirety of these conditions for BMR cannot be met, resting metabolic rate (RMR) is typically used in its place; resting energy expenditure under more natural or flexible conditions (Portugal et al. 2016; Swanson et al. 2017)."

2. I am surprised that the idea that fossorial mammals are supposed to have lower metabolism (suggested by Brian McNab and others).
Apologies, we're not entirely sure what the reviewer is asking or proposing here? Possibly suggesting we should mention the theories and evidence from McNab, that fossorial mammals have lower metabolism? We didn't include this, because more recent work suggests it may be far more complex than that. A recent phylogenetically-informed macroevolutionary study (Merchant et al. 2024) on BMR across 801 species of mammals found no evidence of a signal in BMR associated with a fossorial lifestyle.

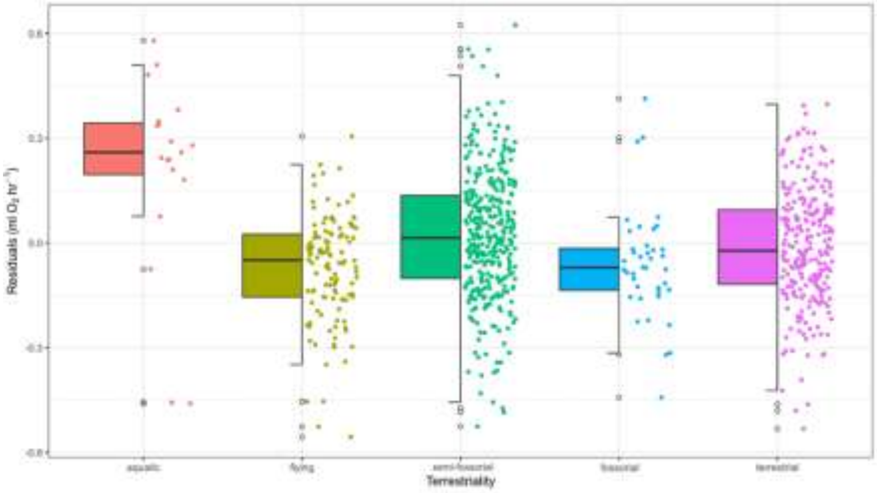

Box and jitter plot showing the residuals of weighted log basal metabolic rate (BMR) (ml O2 h−1) against weighted log body mass (g) across five terrestriality categories for all 801 mammal species used in this study.

Merchant, H. N., Thirkell, J.E. and Portugal, S.J. (2024). No evidence for a signal in mammalian basal metabolic rate associated with a fossorial lifestyle. Sci. Rep. 14, 11297.

3. Metabolism has been measured numerous times in this species, and it would of interest to see a comparison to these earlier measurements. The impact of your paper could be strengthened by the inclusion of tabulated data and a brief discussion of differences/similarities.

© 2026. Published by The Company of Biologists under the terms of the Creative Commons Attribution License (https://creativecommons.org/licenses/by/4.0/).

Thank you for this suggestion. We have now added a Table of the values of RMR from previous studies on naked mole-rats (please see below). It was at times tricky, because of the different units each study used. Some older studies presented individual measures of RMR as ml kg hr^{-1}, but only population level body mass were presented. Thus, for some of the values we had to convert them for comparison to ours, and we had to use a population average body mass to do this. Our values (now additionally presented in Table 1) fall within the previously published ranges.

Table 2. Metabolic measurements from previous studies. Resting metabolic (RMR) and sample size from seven prior studies which reported the RMR of naked mole-rats.

Sample size	RMR (ml O_2 hr^{-1})	Study
14	21.45	McNab 1966
19	42.80	Buffenstein and Yahav 1991
20	36.1	Goldman et al. 1999
4	48.4	Riccio et al. 2000
24	44.2	Connor et al. 2002
22	81.36	Kirby et al. 2018
22	92.4	Parmenter et al. 2019
76	45.5	This Study

The additional text can be found below:

"Colony-level body mass (g), resting metabolic rate (RMR) (ml O_2 hr^{-1}) and respiratory quotient (RQ) data are presented in Table 1. Colony measurements of RMR ranged from 37.9 ± 9.8 ml O_2 hr^{-1} to 52.2 ± 18.9 ml O_2 hr^{-1} (Table 1). Previous values of RMR for naked mole-rats ranged from 21.5 ml O_2 hr^{-1} to 92.4 ml O_2 hr^{-1} (Table 2). Variation in RQ was low within colonies (S.D. range from 0.06 - 0.1) and between colonies, with an average RQ for all individuals (N = 76) of 0.7 ± 0.1 (Table 1)."

4. The authors use intermittent closed respirometry. I think the duration of the flush period and the closed period could be described more firmly (lines 138-140) as 45 min plus 20 min (?).

To clarify, we did not use closed respirometry, explained in the Methods:

"Resting metabolic rate was determined through the measurement of the rate of oxygen consumption ($\dot{V}$O2) and carbon dioxide production ($\dot{V}$CO2), using an open-flow respirometer (Sable Systems International, Las Vegas, NV)".

"Each respirometry assessment lasted a minimum of 65 minutes and consisted of a 10-minute baseline to assess ambient O2 level, a 45-minute metabolic assessment, followed by a further 10-minute baseline to record ambient O2."

As the baseline sessions are 10 minutes either side of the measurements themselves, we felt it would be confusing for the reader if we termed it as 45 minutes plus 20 minutes.

5. The respirometry method must have result in 6-10 measurements per individual. Which was used to reflect SMR and how were these chosen. A typical approach is to choose the lowest measurements by some definition and the idea is to reduce the influence of spontaneous activity as much as possible. As mentioned above, I think the authors underestimate the contribution of spontaneous activity in these very active animals.

This information is provided within the Methods on lines 174-176:

"A 5-minute minimum analysis region was selected for RMR, corresponding to the lowest stable O_2 consumption and CO_2 production, during which the animal was considered to be most restful".

6. The description of the wash-out period is not correct (line 143); it takes much longer as the washout is an exponential and thus linger than volume of the divided relative flow. Normally that value results in 90% washout.

Here, we say:

"The respirometer consisted of an airtight custom-made 2.5L (19.2cm long x 15.2cm wide x 8.8cm high) acrylic container, fitted with 4-mm inlet and outlet ports. Outside air (i.e.,

© 2026. Published by The Company of Biologists under the terms of the Creative Commons Attribution License (https://creativecommons.org/licenses/by/4.0/).

ambient air) was pulled through the respirometer at a flow rate of 300 ml min-1, resulting in a flush-out rate of approximately 8 mins 20 secs"

This refers to a 63% washout rate, and we should have made that clearer and the reviewer is quite correct. Please find our calculations below. This clarity has been added to the manuscript.

Time Constant (τ)

The chamber time constant is calculated as:

$\tau = V / Q$

$\tau = 2500\ mL / 300\ mL\ min^{-1}$

$\tau = 8.33\ min$

Wash-Out Rate Constant (k)

$k = Q / V$

$k = 300 / 2500$

$k = 0.12\ min^{-1}$

Thus:

63%: 8.3 min

95%: 25 min

Thus our 45-minute sampling session of the box when the animal is being assessed is of sufficient time. This information has been added to the manuscript.

7. The authors describe measurements of CO_2 production but this data is not presented. It would be useful if RER was reported in more detail.

Thank you for this suggestion. We have now added a small Table (see below) which presents the RQ data. These data were all available in the supplementary table, but we agree that is useful for these to be presented in the main manuscript, particularly as the RQ values demonstrate that (1) the animals were post-absorptive, and (2) that there was very little variation between individuals and between colonies. We have added a couple of sentences to the Results, to describe this and allude to the Table.

Table 1. Physiological measurements from five colonies of naked-mole rats. For measurement techniques, see Methods. RMR refers to resting metabolic rate (ml O_2 hr^{-1}), and RQ to the respiratory quotient. Values presented are mean ± S.D. RQ is presented with three decimal places due to the small nature of the S.D.

Colony	N	Body mass (g)	RMR (ml O_2 hr^{-1})	RQ
CF27	15	35.9 ± 7.0	37.9 ± 9.8	0.7 ± 0.06
17A	19	33.6 ± 15.0	37.9 ± 15.5	0.7 ± 0.06
G	9	45.8 ± 7.6	52.2 ± 18.9	0.8 ± 0.0.7
B	25	49.2 ± 9.4	50.0 ± 21.4	0.7 ± 0.1
11C	8	35.3 ± 11.9	47.2 ± 26.3	0.7 ± 0.06
All	76	40.8 ± 12.6	45.5 ± 18.8	0.7 ± 0.1

The additional text can be found below:

"Colony-level body mass (g), resting metabolic rate (RMR) (ml O_2 hr^{-1}) and respiratory quotient (RQ) data are presented in Table 1. Colony measurements of RMR ranged from 37.9 ± 9.8 ml O_2 hr^{-1} to 52.2 ± 18.9 ml O_2 hr^{-1} (Table 1). Previous values of RMR for naked mole-rats ranged from 21.5 ml O_2 hr^{-1} to 92.4 ml O_2 hr^{-1} (Table 2). Variation in RQ was low within colonies (S.D. range from 0.06 - 0.1) and between colonies, with an average RQ for all individuals (N = 76) of 0.7 ± 0.1 (Table 1)."

8. Lines 85- (and elsewhere). How different are the colonies in terms of the genetic background. In other words, are differences in metabolism to be expected and why?

We should have provided that. They were all captive born descendants of animals that were originally captured in Kenya during the 1980s and have a mixed genetic background as a result of originally crossing wild caught individuals from the north of Kenya with individuals from the south. This information has now been added to the Methods.

© 2026. Published by The Company of Biologists under the terms of the Creative Commons Attribution License (https://creativecommons.org/licenses/by/4.0/).

9. Line 90. How does this temperature range compare to the thermoneutral zone of this species when kept under the conditions of the measurements (ie solitary with no possibility of huddling etc).
The naked mole rats were kept between 26-32°C, which is within the apparent thermoneutral zone' of 27-34°C (Yahav & Buffenstein 1991). This information has now been added to the manuscript.

Most importantly, however, all individuals measured had been living at these ambient temperatures in the same room and enclosures for a minimum of 5 years (and for many, many more years than that), thus were acclimated to these ambient temperatures.

The reviewer makes a good point, that being solitary may affect this, and that being alone could cause either a stress response, or interact with thermoregulation. Such a problem - measuring metabolic rate in highly social animals alone in a chamber - is not unique to our study. We ensure we make this clear in the manuscript:
"Furthermore, for intrinsically social species, the stress associated with being separated for metabolic assessment has been noted to increase O_2 consumption; Fukomys damarensis exhibited a 5% decrease, per individual, in O_2 consumption when two individuals were assessed together (Wiedenová et al. 2018). It is for these reasons that RMR was considered an appropriate measure of energetic expenditure in this study."

Combined, this is why we call it RMR and not BMR. Moreover, all colonies and individuals were subject to the same conditions and temperatures.

Yahav, S. and Buffenstein, R. (1991). Huddling behaviour facilitates homeothermy in the naked mole rat *Heterocephalus glaber*. Physiol. Zool. 64, 871-884.

10. Lines 94-95. Are there any primary papers to be cited on the duration of the SDA response in mole rats?
Despite extensive searching and communication with various mole rat researchers, we cannot locate any primary papers that refer to, measure, or talk about the SDA response in mole rats.

Reviewer 2:
This study sets out to measure resting metabolic rate in individual naked mole-rats and correlate this to size, age, sex and social status. Five different colonies was studied and quite a lot of work must have gone into these experiments. As the authors point out, the social arrangement of naked mole-rats provides an uncommon framework for separating the effects of body mass from other influences on variation in RMR. The study appears to be professionally conducted and the manuscript is generally well written. However, I do have some comments.
Thank you for your positive comments; they are much appreciated.

The Introduction starts with the statement that "Resting metabolic rate (RMR) represents the baseline level of energy required to sustain essential physiological processes in endothermic animals and forms a central component of an individual's total energy expenditure". Does not that refer to basal metabolic rate (BMR) rather than RMR? For example, on page 5 it is stated that "Resting metabolic rate is considered to be between 10-25% greater than BMR, typically (Sherwood et al. 2005). Thus, the initial statement appears to be contradictory to what is later stated, studied and discussed.
Thank you for highlighting this. You are absolutely correct, and our phraseology is muddled here. We have corrected the terminology. The definitions on page 5 are correct. The updated text can be found below:

"Metabolism is often described as the currency of life (REF). A widely used measure of metabolism is basal metabolic rate (BMR), which represents the minimum amount of energy an endothermic animal requires to sustain essential bodily functions while resting, fasting, non-reproductive, and within its thermal neutral zone (TNZ) (REF). When the entirety of the conditions for BMR cannot be met, resting metabolic rate (RMR) is typically used in its place; resting energy expenditure under more natural or flexible conditions."

© 2026. Published by The Company of Biologists under the terms of the Creative Commons Attribution License (https://creativecommons.org/licenses/by/4.0/).

Page 4. It is stated that "experimental procedures involving live animals and data collection described herein were approved by Royal Holloway University of London and Queen Mary University of London". Journals normally ask for an approval number so that the application can be traced.

Both Royal Holloway and Queen Mary (under the University of London) operate a two-stage system for ethical approval. If, at the first step, the work is not deemed to require Home Office approval, the panel review the project and make recommendations to improve the work, prior to approval. However, they do not provide an approval number, only an email confirming the project can move forward.

Animals: I assume the animals must have been individually marked in some way to allow for the correlations to be made. How was this marking done?

Thank you for pointing out this oversight. Animals were indeed identifiable, through subcutaneous RFID microchips and were marked with black marker pens to allow individuals to be distinguished during observations. This information has now been added to the Methods.

On page 8 it is stated that "Breeding status showed a nonsignificant trend, with non-breeding females having slightly lower RMR ($F_{1,39}$ = 2.89, P = 0.097)". However, in the Abstract it is stated that "Breeding females showed elevated RMR relative to non-breeding females, consistent with reproductive costs.". Similarly, in the Discussion it is said that "Reproductive role had a stronger influence on metabolic rate. Breeding females showed higher RMR than non-breeding individuals". It seems misleading to talk about a "stronger influence" when it was not statistically significant. Please refer to this as a nonsignificant trend (like in the Results) also in the Abstract and Discussion.

We agree that our terminology here is confusing and could be misleading. We have corrected all instances of this, through either removing the statement, or making it clear that this is a nonsignificant trend.

Page 10. It is stated that "Notably the absolute RMR values recorded here are substantially lower than those predicted for mammals of similar size (Kleiber, 1932). Although Kleiber was a pioneer in this field, I suggest that the authors also cite some of the more recent and much more comprehensive papers on this topic.

As suggested, we have broadened out the comparative element here, with more recent comparative studies. We have used 10 studies to demonstrate what the predicted metabolic rate of naked mole-rats would be. These values range from 54.3 ml O_2 hr^{-1} to 71.1 ml O_2 hr^{-1}; the measured average in the present study was 40.8 ml O_2 hr^{-1}.

Table 3. Predicted metabolic rate of naked mole-rats, assuming a body mass of 40g.
Values converted to ml O_2 per hour using the approximation 1 L O_2 ≈ 4.8 kcal.

Reference	kcal/day	ml O_2 hr^{-1}
Rubner (1883)	8.19	71.1
Kleiber (1932)	6.26	54.3
Brody (1945)	6.69	58.1
West et al. (1997)	6.26	54.3
Lovegrove (2000)	5.95	51.6
White & Seymour (2003)	7.87	68.3
Savage et al. (2004)	6.35	55.1
Glazier (2005)	7.12	61.8
McNab (2008)	6.59	57.2
Clarke & Rothery (2010)	6.80	59.0

References

Brody, S. (1945). *Bioenergetics and Growth*. New York, NY: Reinhold Publishing Corporation.

Clarke, A., & Rothery, P. (2010). Scaling of body temperature in mammals and birds. *Functional Ecology*, 24(3), 518-524.

© 2026. Published by The Company of Biologists under the terms of the Creative Commons Attribution License (https://creativecommons.org/licenses/by/4.0/).

Glazier, D. S. (2005). Beyond the '$^{3/4}$-power law': variation in the intra- and interspecific scaling of metabolic rate in animals. *Biological Reviews*, 80(4), 611-662.

Kleiber, M. (1932). Body size and metabolism. *Hilgardia*, 6(11), 315-353.

Lovegrove, B. G. (2000). The zoogeography of mammalian basal metabolic rate. *The American Naturalist*, 156(2), 201-219.

McNab, B. K. (2008). An analysis of the factors that influence the level and scaling of mammalian BMR. *Comparative Biochemistry and Physiology Part A: Molecular & Integrative Physiology*, 151(1), 5-28.

Rubner, M. (1883). *Über den Einfluss der Körpergrösse auf Stoff- und Kraftwechsel. Zeitschrift für Biologie*, 19, 535-562.

Savage, V. M., Gillooly, J. F., Woodruff, W. H., West, G. B., Allen, A. P., Enquist, B. J., & Brown, J. H. (2004). The predominance of quarter-power scaling in biology. *Functional Ecology*, 18(2), 257-282.

West, G. B., Brown, J. H., & Enquist, B. J. (1997). A general model for the origin of allometric scaling laws in biology. *Science*, 276(5309), 122-126.

White, C. R., & Seymour, R. S. (2003). Mammalian basal metabolic rate is proportional to body mass$^{2/3}$. *Proceedings of the National Academy of Sciences*, 100(7), 4046-4049.

Page 11. It is maybe surprising that social rank did not affect RMR. Such effects have been seen in other animals, from fish, to birds and mammals. This may be worth a bit of discussion and citations of studies finding/not finding dominance effects on RMR.

Thank you for this useful suggestion. We have now added a paragraph that discusses this finding in more detail and with greater context, using studies that also did not find a link between RMR and social rank. This new paragraph can be found below:

While it may be somewhat surprising that social rank did not influence RMR in this study, given that dominance-related differences in metabolic rate have been reported across a range of taxa (e.g. fishes, birds, and mammals) controlled (Mathot, Dingemanse and Nakagawa 2019), such effects are not universal. For instance, elevated metabolic rates in dominant individuals have been linked to increased activity, aggression, or the energetic costs of maintaining status in some systems, whereas other studies have found no clear association between dominance and baseline metabolic expenditure once activity and context are controlled (Mathot, Dingemanse and Nakagawa 2019; Senar et al. 2000).

The absence of an effect in the present study may reflect several non-mutually exclusive factors. First, RMR was measured under standardised, resting conditions, which may minimise behavioural or physiological differences associated with social interactions that are more apparent in active metabolic rate. Second, dominance hierarchies in this species/context may be relatively stable or low-cost to maintain, reducing any sustained energetic differences between ranks. Third, environmental conditions (e.g. resource availability, group size, or housing conditions) can modulate the energetic consequences of dominance, potentially weakening rank-metabolism relationships.

Fig. 2. The text and numbers on the axes are very small and hard to read. Please be consistent in the formatting of the figures.

We have now redrawn this Figure so that the text and numbers are larger and more readable. The new Figure can be found below:

© 2026. Published by The Company of Biologists under the terms of the Creative Commons Attribution License (https://creativecommons.org/licenses/by/4.0/).

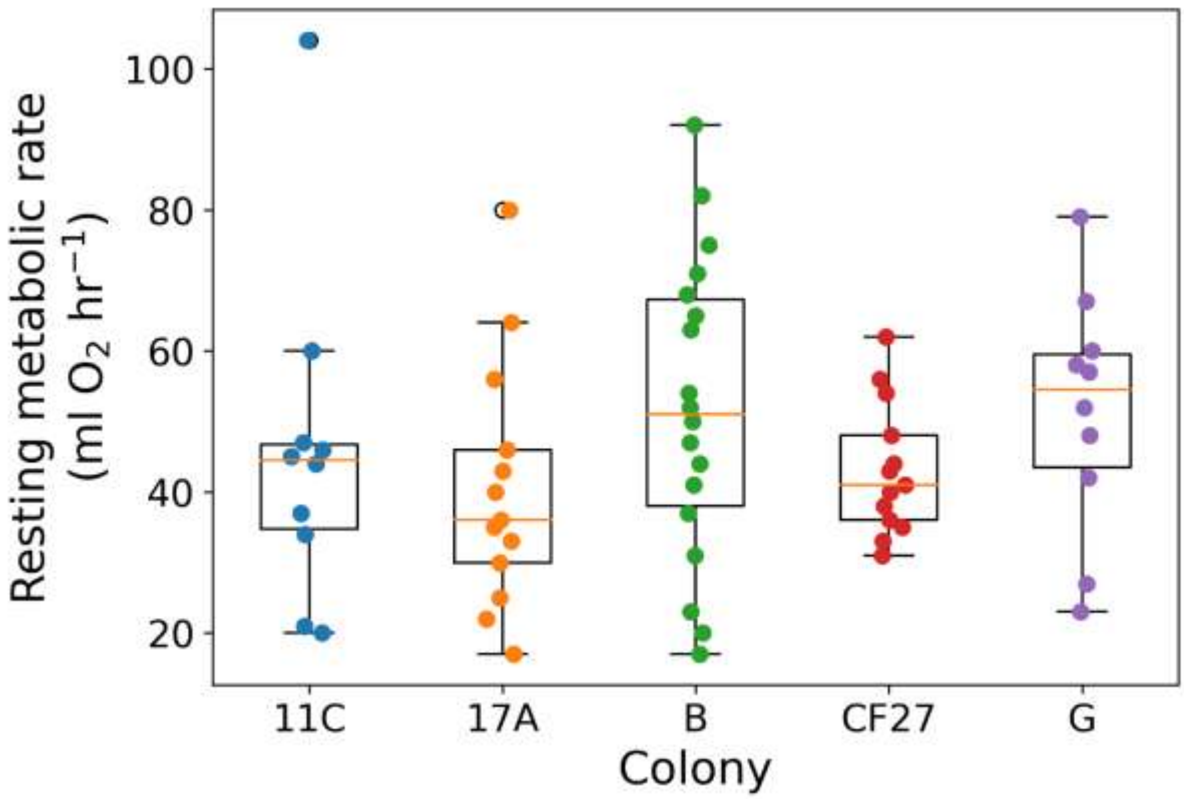

Second decision letter

MS ID#: bio.062586R1

MS Title: Allometric Scaling and Social Modulation of Resting Metabolic Rate in a Eusocial Mammal (Heterocephalus glaber)

Authors: Jack E. Thirkell, Chris G. Faulkes, Hana N. Merchant, James D. Gilbert, Nigel C. Bennett, Daniel W. Hart, Monica A. Daley and Steven J. Portugal

I am happy to tell you that your manuscript has been accepted for publication in Biology Open, pending our standard publication integrity checks. It was accepted on 29th May 2026.

© 2026. Published by The Company of Biologists under the terms of the Creative Commons Attribution License (https://creativecommons.org/licenses/by/4.0/).
